# Prevalence and Associated Risk Factors of Sepsis among Neonates Admitted into Neonatal Intensive Care Units of Public Hospitals in Dhaka

**DOI:** 10.7759/cureus.7461

**Published:** 2020-03-29

**Authors:** Zannatun Nyma, Mahfuzur Rahman, S M Mehedi Hasan, Naym Uddin Roby, Farhana Khanam, Md Ehsanul Alam, Mohammad Ali

**Affiliations:** 1 Epidemiology and Public Health, American International University Bangladesh, Dhaka, BGD; 2 Epidemiology and Public Health, International Centre for Diarrhoeal Disease Research, Dhaka, BGD; 3 Epidemiology and Public Health, United International University, Dhaka, BGD; 4 Epidemiology and Public Health, North South University, Dhaka, BGD; 5 Physical Medicine and Rehabilitation, Dhaka Medical College and Hospital, Dhaka, BGD

**Keywords:** neonatal sepsis, prevalence, risk factors, bangladesh

## Abstract

Objective: To determine the prevalence and associated risk factors of sepsis among neonates admitted into neonatal intensive care units (NICU) of public hospitals in Dhaka.

Methods: This was a cross-sectional study conducted among 173 neonates admitted into the NICUs of Dhaka Medical College Hospital (DMCH) and Dhaka Shishu (Children) Hospital from March 1, 2016 to September 30, 2016 at Dhaka, Bangladesh. On the basis of the presence of clinical signs and symptoms of sepsis, neonates were admitted into the NICUs. The weight of the baby was measured and blood culture, complete blood count (CBC), C-reactive protein (CRP) and urine R/M/E were done at the time of admission. The neonates, who had positive blood culture reports, were confirmed as having sepsis. After receiving informed written consent, maternal data were collected from the mother of the neonate and neonatal data were collected from NICUs.

Results: The prevalence of sepsis among the neonates admitted into NICU of the concerned public hospitals in Dhaka was 69.35%. In the multiple logistic regression model, perinatal asphyxia (adjusted odds ratio (aOR) = 3.37, 95% confidence interval (CI) = 1.27-8.90), presence of infection at umbilical cord (aOR = 3.32, 95% CI = 1.40-7.85), history of bottle feeding of the neonates (aOR = 3.02, 95% CI = 1.11-8.25) and pre-existing maternal infection (aOR = 4.44, 95% CI = 1.92-10.26) were significantly (p-value < 0.05) associated with neonatal sepsis. The odds of developing sepsis among the neonates with ≤ 2.5 kg weight at admission was more than three times higher (aOR 3.82, 95% CI = 1.59-9.19) than neonates with admission weight > 2.5 kg.

Conclusion: Like other South Asian countries, the prevalence of neonatal sepsis is alarming in Bangladesh. Further research should be conducted to measure the burden of infections in the entire neonatal period and observe the effects of biological risk factors on the early and late-onset neonatal sepsis.

## Introduction

Over four million neonates die each year globally with the majority of the deaths occurring in low- and middle-income countries (LMICs) [1,2]. The estimated neonatal mortality rate in LMICs is 20 per 1000 live births, compared to three per 1000 in high-income countries [2]. These deaths have been categorically attributed to neonatal sepsis, meningitis, respiratory tract infections, diarrhoeal diseases, neonatal tetanus and prematurity which, in most cases, are preventable or treatable [3,4]. Previous studies revealed neonatal sepsis to be the most common cause of neonatal mortality [5,6].

Neonatal sepsis, a systemic infection precipitating within the first 28 days after birth, encompasses blood-stream infection, meningitis, and pneumonia [7]. It currently is responsible for about 1.6 million annual deaths among neonates worldwide, 99% of which take place in developing countries [8,9]. Of the total sepsis-related neonatal deaths in 2013, 38.9% occurred in South Asia alone [7]. Epidemiological estimates suggested that the global prevalence of neonatal sepsis was 1.7 million in 2010 [10]. South Asia and sub-Saharan African countries share the highest-burden of neonatal sepsis cases in the world; Bangladesh, being a developing South Asian country, is not an exception to this very trend [7,11].

Neonatal sepsis is a high-priority public health issue, particularly in the context of a developing country, where it constantly emerges as one of the major contributors to neonatal morbidity and mortality [12]. In addition to the substantial percentage of immediate mortality, neonatal sepsis imposes a wide range and varying degrees of long-term disabilities upon the survivors in their future [13]. Previous research revealed that such long-term impairments might be in the form of cognitive disability, learning disabilities, developmental delays, hearing loss or visual disturbance, resulting in a major socio-economic burden in resource-poor countries and contributing to the global disease burden [14,15]. The global burden of morbidity was estimated at about 3% of all Disability Adjusted Life Years (DALYs) [16]. Moreover, neonatal sepsis leads to the causes of neonatal hospital admissions in developing countries [17].

Some studies conducted in Bangladesh focused only on the bacteriological profile and antimicrobial susceptibility regarding neonatal sepsis [18-20]. As neonatal sepsis encompasses a number of diseases that are preventable, along with lab-based study for causative organisms, it is crucial to identify the risk factors related to it [21]. Depending on the variation of the study population, marked divergence concerning risk factors of neonatal sepsis has been reported [12]. In Bangladesh, only very few studies attempted to determine the risk factors of neonatal sepsis [22-23]. The overall lack of data on neonatal sepsis in Bangladesh spurred this research to identify risk factors influencing neonatal sepsis so that appropriate intervention measures and resource mobilization can be initiated for addressing the modifiable risk factors. Identification of the risk factors associated with neonatal sepsis could also provide significant insights leading to new findings for neonatal sepsis prevention, early diagnosis, and better treatment, thereby reducing morbidity and mortality.

Considering all these contexts, we carried out a cross-sectional study to determine the prevalence and associated risk factors of sepsis among neonates admitted into neonatal intensive care units (NICU) of public hospitals in Dhaka.

## Materials and methods

We conducted an institution based cross-sectional study in the NICUs of Dhaka Medical College Hospital (DMCH) and Dhaka Shishu (Children) Hospital. The study was undertaken from March 1, 2016 to September 30, 2016 and included 173 neonates admitted in the NICUs of these two hospitals during the study period.

The NICUs of DMCH and Dhaka Shishu (Children) Hospital had the capacity to treat 36 and 14 neonates respectively at a time during the study period. The weight of the baby was measured at the time of admission. In suspected neonatal sepsis cases, physicians admitted the neonates into the NICUs of the hospitals on the basis of the presence of clinical signs and symptoms of neonatal sepsis [24]. Blood culture, complete blood count (CBC), C-reactive protein (CRP) and urine R/M/E were done at the time of admission. The neonates, who had positive blood culture reports, were confirmed as having sepsis. Inclusion criteria were - all neonates (0-28 days) admitted in the particular NICUs during the study period and whose mothers gave informed consent to include her baby in our study. Exclusion criteria included - any case of neonatal death taking place in the concerned ICUs during the data collection period and neonates whose mothers refused to give informed consent.

A standard semi-structured questionnaire was used to collect neonatal and maternal data. Before data collection, the mother of a neonate was given a detailed explanation of the study. After receiving informed written consent, maternal data were collected from the mother of the neonate which included socio-demographic conditions, location of delivery, maternal health conditions. Neonatal data were collected from NICUs which included admission weight of the baby, presence of neonatal sepsis, the gender of the baby, TORCH infection (toxoplasmosis, others - syphilis, rubella, cytomegalovirus, and herpes simplex viral infection), perinatal asphyxia, fetal malformation, cord care of the neonate, history of bottle feeding and pre-lacteal feeding. The presence of neonatal sepsis was the outcome variable. Neonatal sepsis and perinatal asphyxia were defined as follows:

Neonatal sepsis was defined as a systemic infection occurring in the first 28 days of life that encompassed blood-stream infection, meningitis and pneumonia occurring among the neonates evidenced by positive blood culture report [7].

Perinatal asphyxia is a clinical condition that results from the impaired gas exchange in the fetus. This leads to hypoxia, hypercarbia, and acidosis and these ultimately culminate in failure to establish and sustain spontaneous respiration immediately after birth [24].

After gathering all the required information, data was compiled, analyzed and tabulated in accordance with key variables. Data analysis was performed on the basis of the research question and the objective. The statistical software package Stata, version 14.0 (LP StataCorp, College Station, TX) was used to analyze the data. Descriptive statistics were calculated for all the variables, including mean, standard deviation, frequencies, and percentages. In order to assess the association between the outcome variable (neonatal sepsis) and independent variables, at first, we performed a simple logistic regression analysis (Chi-square test). The variables that were found statistically significant (having p-value ≤ 0.25) at the simple logistic regression model, we kept them in a multiple logistic regression model. The results of the multiple logistic regression model were presented in terms of the adjusted odds ratio (aOR) with respective 95% confidence interval (CI).

Ethical approval for our study protocol was obtained from the Ethical Review Committee of the American International University Bangladesh (AIUB). Permission for collecting data was taken from the authority of DMCH and Dhaka Shishu (Children) Hospital.

## Results

**Figure 1:**
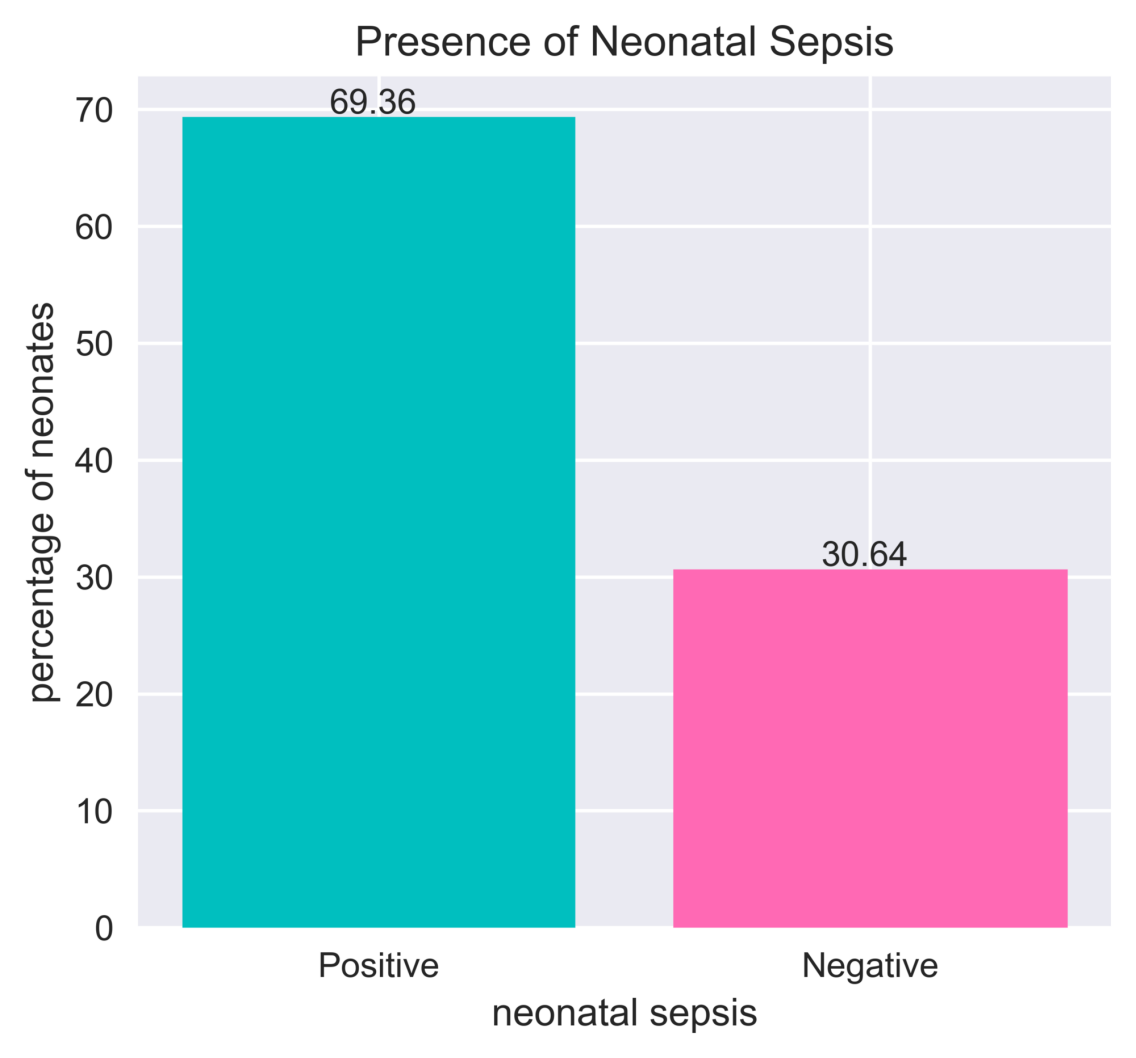
Presence of neonatal sepsis

A total of 173 neonates were included in our study, of which 69.36% (120 neonates) had been suffering from neonatal sepsis (Figure 1). Admission weight of 124 neonates was ≤ 2.5 kg and mean admission weight was 1.28 (standard deviation (SD)=0.45). Fifty-eight percent of neonates were male and 79.77% had been suffering from perinatal asphyxia. Sixty-six percent of neonates represented the presence of infection at the umbilical cord.

The mean age of the mothers of the neonates was 24.81 (SD=5.47) and mean age at the marriage of them was 19.93 (SD=3.27). Fifty-five percent of mothers did not complete secondary school certificate (SSC) level and 67.63% of them were unemployed. Among 173 mothers, 63 had a history of single unclear or >3 sterile vaginal examinations during labor. Fifty-five percent of mothers had pre-existing maternal infections (TORCH infection/ hepatitis B, hepatitis C/ hepatitis E/ gonorrhea/chlamydia). Among 173 mothers, 135 had the history of co-morbidities state (pre-eclampsia/ diabetes/ urinary tract infection (UTI)/ pneumonia/maternal peripartum fever) (Table 1).

**Table 1:** Study population characteristics SSC: Secondary school certificate; SD: standard deviation.

Variables	Values
Maternal Characteristics	
Age in years (mean ± SD)	24.81±5.47
Age at marriage in years (mean ± SD)	19.93±3.27
Academic qualification (%, N)	
≤ SSC Level	55.49 (96)
Occupational status (%, N)	
Unemployed	67.63 (117)
Family income (Per month) (%, N)	
≤15000	62.43 (108)
Location of delivery (%, N)	
Hospital	83.24 (144)
Home	15.61 (27)
Others (on the way to hospital, on footpath-police case)	1.16 (2)
Premature rupture of membrane (PROM) (%, N)	39.31 (68)
Anti-partum haemorrhage (APH) (%, N)	26.59 (46)
Multiple order of pregnancy (%,N)	45.09 (78)
Single unclear or >3 sterile vaginal examinations during labor (%, N)	
Yes	36.42 (63)
No	46.82 (81)
Not Sure	16.76 (29)
Pre-existing maternal infection (%, N)	
Yes	54.91 (95)
No	45.09 (78)
Co-morbid states (%, N)	
Yes	78.03 (135)
No	21.97 (38)
Anemia (%, N)	
<11.0g/dlor6.8mmol/L	62.43 (108)
Neonatal Characteristics	
Admission weight (%, N)	
≤2.5 kg	71.68 (124)
>2.5 kg	28.32 (49)
Gender (%, N)	
Male	57.8 (100)
Female	42.2 (73)
Perinatal Asphyxia (%, N)	
Present	79.77 (138)
Absent	20.23 (35)
Presence of infection at umbilical cord (%, N)	
Yes	65.32 (113)
No	34.68 (60)
History of bottle feeding (%, N)	34.1 (59)
History of pre-lacteal feeding (%, N)	14.45 (25)

We presented the association of neonatal sepsis with different independent variables such as admission weight, gender, perinatal asphyxia, cord care, history of bottle feeding of the neonates, pre-existing maternal infection, co-morbid states, UTI, anemia and single unclear or >3 sterile vaginal examinations during labor in bivariate analysis (Table 2).

**Table 2:** Association between neonatal sepsis and independent variables (unadjusted)

Variable label	Category	Neonatal sepsis		p-value
		Present (%, n)	Absent (%, n)	
Maternal Characteristics				
Single unclear or >3 sterile vaginal examinations during labor	Yes	79.37 (50)	20.63 (13)	0.031
	No	63.64 (70)	36.36 (40)	
Pre-existing maternal infection	No	52.56 (41)	47.44 (37)	<0.001
	Yes	83.16 (79)	16.84 (16)	
Co-morbid states	No	55.26 (21)	44.74 (17)	0.033
	Yes	73.33 (99)	26.67 (36)	
Urinary tract infection (UTI)	No	64.81 (35)	35.19 (19)	0.068
	Yes	79.01 (64)	20.99 (17)	
Anemia	<11.0g/dlor6.8mmol/L	76.85 (83)	23.15 (25)	0.006
	>11.0g/dlor6.8mmol/L	56.92 (37)	43.08 (28)	
Child Characteristics				
Admission weight	≤ 2.5 kg	81.45 (101)	18.55 (23)	<0.001
	> 2.5 kg	38.78 (19)	61.22 (30)	
Gender	Male	73 (73)	27 (27)	0.225
	Female	64.38 (47)	35.62 (26)	
Perinatal Asphyxia	Yes	76.81 (106)	23.19 (32)	<0.001
	No	40 (14)	60 (21)	
Presence of infection at umbilical cord	Yes	83.19 (94)	16.81 (19)	<0.001
	No	43.33 (26)	56.67 (34)	
History of bottle feeding	Yes	83.05 (49)	16.95 (10)	0.005
	No	62.28 (71)	37.72 (43)	

In the multiple logistic regression model, admission weight, perinatal asphyxia, presence of infection at the umbilical cord, history of bottle feeding of the neonates and pre-existing maternal infection were significantly (p-value < 0.05) associated with neonatal sepsis (Table 3).

**Table 3:** Association between neonatal sepsis and independent variables (adjusted)

Variable label	Category	Odds Ratio (adjusted)	p-value	95% CI
Maternal Characteristics				
Pre-existing maternal infection	No	Reference		
	Yes	4.44	<0.001	(1.92, 10.26)
Child Characteristics				
Admission weight	> 2.5 kg	Reference		
	≤ 2.5 kg	3.82	0.003	(1.59, 9.19)
Perinatal Asphyxia	No	Reference		
	Yes	3.37	0.014	(1.27, 8.90)
Presence of infection at umbilical cord	No	Reference		
	Yes	3.32	0.006	(1.40, 7.85)
History of bottle feeding	No	Reference		
	Yes	3.02	0.031	(1.11, 8.25)

The neonates whose mothers had the pre-existing infection had more than four (aOR = 4.44, 95% CI = 1.92-10.26) times higher chance of developing neonatal sepsis compared to the neonates whose mothers did not have pre-existing infections. The odds of developing sepsis among the neonates with ≤ 2.5 kg weight at admission was more than three times higher (aOR 3.82, CI: 1.59 to 9.19) than neonates with admission weight > 2.5 kg. Neonates having perinatal asphyxia had 3.37 times (CI: 1.27 to 8.90) higher chance of developing neonatal sepsis compared to those who did not have this complaint/ diagnosis. Bottle-fed neonates had 3.02 times (CI: 1.11 to 8.25) higher chance of developing sepsis. The adjusted odds of developing neonatal sepsis among children with umbilical cord infection were 3.32 times higher compared to neonates without cord infection.

## Discussion

This study illustrates the prevalence of neonatal sepsis which is one of the prime causes of hospitalization for neonates in developing countries [25-26]. This study also revealed the association of risk factors with sepsis after the hospitalization of neonates. We categorized maternal and neonatal factors to see their relationship with sepsis. Maternal factors mainly include urinary tract infection, pre-existing maternal infection and anemia while neonatal factors include admission weight, perinatal asphyxia, presence of infection at umbilical cord and history of bottle feeding of the neonates.

Our study found a high prevalence of neonatal sepsis (69.36%) in NICU admitted patients. Another study at Chittagong Medical College Hospital, Bangladesh had almost the same findings of early-onset neonatal sepsis (65.38%) and late-onset neonatal sepsis (34.62%) [18]. Although the findings appeared similar, the study from Motara et al. showed about 5% early-onset and 91% late-onset neonatal sepsis [25]. In Indian studies, the prevalence of neonatal sepsis was found 23.3% in Bihar which was lower than other cities like Delhi [26,27]. This could be due to the higher socio-demographic, economic or education level. A community-based study proved a higher incidence (14.5%) of sepsis in neonates in Bangladesh [28].

In this study, we found that the mothers’ educational level below secondary level had a higher effect in neonatal sepsis which is similar to another study from Saqeeb et al. [29]. Several risk factors are associated with early and late-onset of neonatal sepsis and low birth weight was found highly significant [27-29]. We found that almost 81.5% of patients in NICU were suffering from sepsis and their admission weight was 2.5kg. Some studies indicated, perinatal asphyxia as one of the major reasons for developing sepsis in neonates, and this is similar to our study [12,27,29]. The study from Mitra et al. found that uncleaned cord care had a significant effect on developing sepsis in neonates [28]. In our observation, there was a higher proportion of umbilical cord infection which supports other studies.

Some other factors like history of bottle feeding of the neonates, pre-existing maternal infection were also found responsible for developing sepsis in neonates. Though UTI had a significant association with developing neonatal sepsis, our research did not find any significance [30]. There should be further prospective research implemented with a larger sample size to observe the effects of biological risk factors on early- and late-onset neonatal sepsis.

Turning to our limitations, some biologically significant variables related to maternal characteristics such as academic qualification, area of residence, premature rupture of membrane (PROM), history of co-morbid state (pre-eclampsia, gestational diabetes mellitus, UTI, pneumonia) and anemia were represented as statistically insignificant because of the small sample size (173 neonates) in our study. Moreover, we could not follow the neonates from birth (16% of mothers participated in our study had history of home delivery and 1.16% had history of delivery on the way to hospital and footpath), so we were unable to take birth weight of the neonates.

## Conclusions

The prevalence of neonatal sepsis among the neonates admitted at NICU in Bangladeshi public hospitals is higher. Major determinants of neonatal sepsis are pre-existing maternal infection, weight of the baby, perinatal asphyxia, and presence of infection at the umbilical cord of the neonate. Therefore, we recommend interventions at three stages - during pregnancy, during delivery, and during neonatal period in order to address the problem of neonatal sepsis. However, further studies are needed to be conducted with large sample size to strengthen our observation.
